# Preferential activation of HIF-2α adaptive signalling in neuronal-like cells in response to acute hypoxia

**DOI:** 10.1371/journal.pone.0185664

**Published:** 2017-10-02

**Authors:** Miguel A. S. Martín-Aragón Baudel, Mick T. Rae, Mark G. Darlison, Amy V. Poole, Jennifer A. Fraser

**Affiliations:** School of Applied Sciences. Edinburgh Napier University, Sighthill Campus, Edinburgh, United Kingdom; University of Thessaly Faculty of Medicine, GREECE

## Abstract

Stroke causes severe neuronal damage as disrupted cerebral blood flow starves neurons of oxygen and glucose. The hypoxia inducible factors (HIF-1α and HIF-2α) orchestrate oxygen homeostasis and regulate specific aspects of hypoxic adaptation. Here we show the importance of HIF-2α dependant signalling in neuronal adaptation to hypoxic insult. PC12 and NT2 cells were differentiated into neuronal-like cells using NGF and retinoic acid, and exposed to acute hypoxia (1% O_2_). Gene and protein expression was analysed by qPCR and immunoblotting and the neuronal-like phenotype was examined. PC12 and NT2 differentiation promoted neurite extension and expression of neuronal markers, NSE and KCC2. Induction of HIF-1α mRNA or protein was not detected in hypoxic neuronal-like cells, however marked induction of HIF-2α mRNA and protein expression was observed. Induction of HIF-1α target genes was also not detected in response to acute hypoxia, however significant induction of HIF-2α transcriptional targets was clearly evident. Furthermore, hypoxic insult dramatically reduced both neurite number and length, and attenuated expression of neuronal markers, NSE and KCC2. This correlated with an increase in expression of the neural progenitor and stem cell-like markers, CD44 and vimentin, suggesting HIF-2α molecular mechanisms could potentially promote regression of neuronal-like cells to a stem-like state and trigger neuronal recovery following ischaemic insult. Our findings suggest the HIF-2α pathway predominates over HIF-1α signalling in neuronal-like cells following acute hypoxia.

## Introduction

Blockage of cerebral arteries starves neurons of oxygen and glucose, triggering a cascade of events leading to irreversible cell death [1]. The tissue surrounding the blockage is partially perfused, therefore neurons here are vulnerable, yet salvageable, and must adapt to survive, to prevent further loss of neuronal tissue [2].

The hypoxia inducible factors (HIFs) are master regulators of oxygen homeostasis and critical for adaptation to hypoxic insult [3]. The HIF alpha subunit exist as three isoforms; HIF-1α, -2α and -3α. HIF-1α and 2α are structurally similar and share common transcriptional targets, including *Slc2A1* and *VEGF* [4,5]. HIF-1α and -2α also regulate distinct subsets of genes and elicit different cellular fates. *HIF-1α* regulates *Pdk* and *LdhA* expression to maintain metabolism, and can activate *Bnip3* to trigger apoptosis, whilst *HIF-2α* promotes angiogenesis, cell division and tissue regeneration by regulating the expression of *EPO*, *Cyclin D1* and the stem cell marker, *Oct4*, respectively [4,6]. By contrast, HIF-3α exists in several alternatively spliced forms and may negatively regulate HIF-1α and -2α [7–9].

There has been considerable interest in the importance of HIF regulated pathways in the pathogenesis of stroke yet their role in stroke pathophysiology, particularly *HIF-1α*, is controversial. Targeted knockout of neuronal *HIF-1α* increased ischaemic damage, infarct volume and mortality following transient cerebral artery occlusion [10], whilst indirect induction of *HIF*, via genetic ablation of *Phd*2, reduced infarct size and improved sensorimotor function following transient ischaemia [11], suggesting *HIF-1α* mediated adaptation may be neuroprotective. However, neuronal-specific knockdown of *HIF-1α* and *HIF-2α* expression was shown to decrease infarct size and improve neuronal survival in the early acute stages of middle cerebral artery occlusion [12], suggesting *HIF* signalling could contribute to stroke-associated damage.

*HIF-1α* and *-2α* display temporal differences in signalling[6]; *HIF-1α* appears to be involved in adaptation to acute hypoxia whilst *HIF-2α* mediates adaptation to chronic hypoxic stress [13]. The timescale of HIF signalling may therefore be critical for effective recovery from stroke. Indeed, whilst ablation of neuronal *HIF-1α* and *HIF-2α* is reported to be beneficial in the hours following stroke, loss of *HIF-1α* and *-2α* correlated with increased apoptosis and reduced sensorimotor function in later stages [12]. This may be due to the importance of angiogenesis in stroke-associated neurogenesis [14,15] and *HIF’s* key role in tuning *VEGF* signalling and angiogenesis [5,16]. These studies highlight the intricacies of HIF signalling, and their potential importance in neuroprotection and recovery from stroke damage.

Adaptation is central to neuronal recovery and stroke repair, however therapies promoting neuronal repair and regeneration are currently lacking. Fully understanding the adaptive mechanisms triggered in response to stroke is essential to develop novel therapeutics to enhance neuronal repair and regeneration, and limit the damage and disability associated with stroke [17].

In this study, neuronal cell lines were used as a model to study the molecular changes occurring in response to acute hypoxic stress. We observed preferential activation of HIF-2α dependant adaptive mechanisms in neuronal-like cells in response to acute hypoxia and an absence of HIF-1α dependant signalling. We also observed increased expression of neural progenitor stem cell-like markers, thought to be transcriptionally regulated by HIF-2α. Together, these findings underscore the importance of HIF-2α signalling in neuronal adaptation following acute hypoxic stress and highlight the potential for neuronal repair and regeneration.

## Experimental procedures

### Cell culture

PC12, NT2 and MCF7 cell lines were obtained from the American Type Culture Collection (ATCC). NT2 and MCF7 cells were maintained in Dulbecco’s Modified Eagle’s Media (DMEM, Gibco) supplemented with 10% (v/v) heat-inactivated foetal bovine serum (FBS, Sigma) and 1% (v/v) penicillin-streptomycin (Sigma). PC12 culture media was also supplemented with 5% (v/v) heat-inactivated horse serum (Sigma). Cells were grown at 37°C in 5% (v/v) CO_2_ atmosphere under high humidity.

### PC12 and NT2 differentiation

PC12 cells were cultured on poly-L-lysine (0.1 mg/mL, Sigma) coated 6-well plates at a density of 2x10^6^ cells/well. After 24 hours, media was replaced with differentiation media (200 nM nerve growth factor (NGF; Sigma, Cat no: N0513), 1% (v/v) horse serum (Sigma) and 1% (v/v) penicillin-streptomycin in DMEM), and replenished every 2–3 days. NT2 cells were differentiated into a neuronal population by the method by Pleasure *et al*., (1992)[18]. Briefly, cells were seeded at 1 x10^6^ in a T75 flask and grown in complete culture media containing all-trans-retinoic acid (ATRA, Sigma, 10 μM); media was replaced twice per week. After 4 weeks of ATRA treatment, NT2 cells were sub-cultured and grown for a further 2 weeks in media containing cytosine arabinoside (1 μM) and fluorodeoxyuridine (10 μM); media was replenished every 3–4 days. Cell morphology was analysed via bright field microscopy and images were captured using an inverted microscope and camera (Zeiss, Primovert and Axiocam) at 20x magnification.

#### AlamarBlue

Cells were seeded at 10^5^ cells/well in a 96-well plate. After 24 hours, media was removed and 100 μL fresh media was added per well. AlamarBlue (ThermoFisher) was added to a final concentration of 10% (v/v). Negative controls contained media only without cells. Absorbance was measured at 550 nm and 600 nm using a plate reader (Elisa Reader LT-5000MS, LabTech International Ltd., Uckfield, UK) and data analysed using Manta software. Normoxic cells were taken to represent 100% mitochondrial activity and results were expressed as a percentage of mitochondrial activity compared to normoxic conditions.

#### Trypan blue staining

Cell number was determined using a Neubauer bright line haemocytometer and trypan blue (Sigma) staining. Following trypsinisation, cells were resuspended in complete media and diluted 1:1 (v/v) with trypan blue. The number of viable and non-viable cells were counted using a haemocytometer and the average viable:non-viable ratio was recorded as the total viable cells/mL. The percentage of viable cells (%) was calculated as: [1.00 –(number of trypan blue positive cells/number of total cells)] × 100.

### Experimental hypoxia

Acute hypoxia was induced in PC12 and NT2 cells following differentiation. MCF7 cells were seeded 24 hours prior to the induction of hypoxia at a density of 2 x10^6^ cells/well in a 6 well dish. Cells were placed in a modular hypoxic chamber (Billups-Rothenberg, Inc.) and hypoxia was induced following the method by Wu and Yotnda (2011)[19]. Briefly, the chamber was sealed and flushed with 1% O_2_ gas mixture at a flow rate of 20 L/minute for 10 minutes then incubated at 37°C in a 5% CO_2_ incubator. After 1 hour, the chamber was purged again with 1% O_2_ gas mixture at 20 L/minute for a further 10 minutes then incubated at 37°C in a 5% CO_2_ incubator for the remainder of the experimental exposure. The pH of the media remained stable using these hypoxic conditions.

### RNA extraction and analysis

Cells were harvested on ice by scraping into ice cold PBS and collected by centrifugation at 4°C at 3000 rpm in a benchtop centrifuge. RNA was extracted using Tri reagent (Ambion) at a ratio of 1 mL per 1x10^6^ cells following the manufacturer’s instructions. RNA concentrations were determined using a NanoDrop spectrophotometer (ThermoFisher Scientific). RNA purity and integrity was assessed by Bioanalyzer (Agilent) and only samples with an RNA integrity number (RIN) >7 were used for downstream analysis. cDNA was synthesised using the nanoScript 2 Reverse Transcription premix kit (Primer Design) according to the manufacturer’s instructions using a thermal cycler (Applied Biosystems Light Cycler 480).

### Quantitative real-time PCR (qPCR)

Oligonucleotides were obtained from MWG Eurofins (UK) and are listed in Table 1. qPCR reactions (20 μL) containing 300 nM oligonucleotides, 1X PrimerDesign Precision qPCR Mastermix, and 25 ng of cDNA in RNAse/DNAse free DEPC-H_2_O. Controls included reactions containing cDNA from a reaction without reverse transcriptase (negative control) and cDNA replaced with nuclease-free water (template negative). Reactions were performed in triplicate using a StepOne^™^ Real Time PCR system (Applied Biosystems) under standard conditions and analysed using StepOne^™^ Software, V2.2. Stable reference genes were identified via Genorm analysis (PrimerDesign, UK) from a panel of 12 human or rat reference genes. Gene stability was calculated with *qbase+* software (Biogazelle). Expression of target genes were analysed relative to topoisomerase (*TOP1*) in PC12 cells and beta actin (*ACTB*) in NT2 and MCF7 cells and quantified using the 2–[delta][delta]Ct method [20].

**Table 1:** The forward (F) and reverse (R) oligonucleotides used in this study.

Target gene	Sequence 5’-3’	Species specificity
***CA9***	F:AGGGTGTCATCTGGACTGTG, R:TGTGTGGCTCGGAAGTTCAG	Human/Rat
***Caspr1***	F:TGACTCTGAACTTGGAGGGTCGTG, R:TATAGCGCATCCATGTGCCAGTCT	Human/Rat
***CD44***	F:GGATCAGGCATTGATGATGATGA, R:TTGGGTTCCACTGGGTCC	Human/Rat
***CHOP***	F: AGCTGGAAGCCTGGTATGAGG, R: GTGCTTGTGACCTCTGCTGG	Human/Rat
***Grp78***	F:TATGGTGCTGCTGTCCAGG, R:CTGAGACTTCTTGGTAGGCAC	Human/Rat
***HIF-1α***	F:GTACCCTAACTAGCCGAGGAAGAA, R:GTGAATGTGGCCTGTGCAGT, F:GCATCTCCACCTTCTACCC, R:CTCTTTCCTGCTCTGTCTG,	Human [21], Rat [22]
***HIF-2α***	F:ACCTGGAAGGTCTTGCACTGC, R:TCACACATGATGATGAGGCAGG	Human/Rat
***HIF-3α***	F:AGGATTGCAGAAGTGGCTGG, R:ATACTGCCCTGTTACTGCCTG	Human/Rat
***NEFH***	F:AGGAGTGGTTCCGAGTGAG, R:GGAGATAACTGAGTACCGGC	Human/Rat
***Nrn1***	F:GCATCTGGTGAATAATCGCTCACG, R:ACTGAAGGAGGCGACGACAATAGC	Human/Rat
***PDI***	F:TGCCCAAGAGTGTGTCTGAC, R:CTGGTTGTCGGTGTGGTC	Human/Rat
***Ptbp2***	F:TTTGTCCGGTTCGGCAATGG, R:GGACTACTGAGAACACTGCCTG	Human/Rat
***SLC2A1***	F:GCTGTGCTTATGGGCTTCTC, R:CACATACATGGGCACAAAGC, F:CCTTGCCTGAGACCAGTTGAA, R:ACAGCAGGGCAGGAGTGTC	Human, Rat [23]
***SLC2A3***	F:CAATGCTCCTGAGAAGATCATAAAGG, R:GAATTGCGCCTGCCAAAG, F:CGCCTGATTATTGGCATCTT, R:TCCAAACCAAAGACCTGAGC	Human, Rat [24]
***Tmod1***	F:GCTCTTGCTGAAATGCTGAA, R:AAGGCTGGCTCTGGTTGTC	Human/Rat
***Vimentin***	F:AGATTCAGGAACAGCATGTCC, R:AGCCTCAGAGAGGTCAGC	Human/Rat

### Immunostaining

Cells were lysed in NP-40 lysis buffer (50 mM Tris, pH 8, 150 mM NaCl, 5mM EDTA, 1% NP-40) containing 1X Halt^™^ protease inhibitor cocktail (ThermoFisher) for 30 minutes on ice and clarified by centrifugation at 4°C at 13000 rpm for 5 minutes. Protein concentrations were estimated by bicinchoninic acid assay (BCA) analysis[25] and samples were prepared in reducing Laemmli buffer (Sigma) and boiled before use. Twenty micrograms of cell lysate was resolved via 8, 10 or 12% SDS-polyacrylamide gel, transferred to 0.45 μm nitrocellulose membrane (Millipore) and blocked with 5% (w/v) non-fat milk (Marvel) in TBS-tween (0.1% v/v) for 1 hour. Protein expression was analysed via immunoblotting using anti-NSE (AbCam, #AB16808), anti-KCC2 (Merck Millipore, #07–432), anti-actin (Santa Cruz, #SC-1615), anti-HIF1α (BD Sciences, #610958; Abcam, #ab1), anti-HIF-2α (1:500, R&D Systems, #AF2886), anti-HIF-3α (Acris antibodies, #AP20606PU-N), anti-CD44 (CST, #5640) or anti-Vimentin (BD Sciences, #550513) at a dilution of 1:1000 in 3% non-fat milk in 1x TBS-tween (0.1% v/v). Anti-goat 680LT (LI-COR, #925–68024), anti-rabbit 680LT (LI-COR, #925–68021) and anti-rabbit 800 CW (LI-COR, #925–32280) IRDye conjugated secondary antibodies, were used at a dilution of 1:10000 in 5% (w/v) non-fat milk in 1x TBS-tween (0.1% v/v) containing 0.01% SDS. Membranes were imaged using a LI-COR Odyssey imaging system (LI-COR, Cambridge, UK) and analysed using Image Studio v2.0.

### Statistical analysis

All analyses were conducted using GraphPad Prism v7.0 (GraphPad Software Inc). Results are shown as the mean ± SEM, where n = 3. For single comparisons, significance was determined using unpaired Student’s *t-test*; for multiple comparisons relative to untreated values, significance was determined using ANOVA with Dunnett's correction; *p<0.05; **p≤0.01; ***p≤0.001.

## Results

### Characterising the *in vitro* changes associated with the neuronal-like phenotype

**Fig 1:**
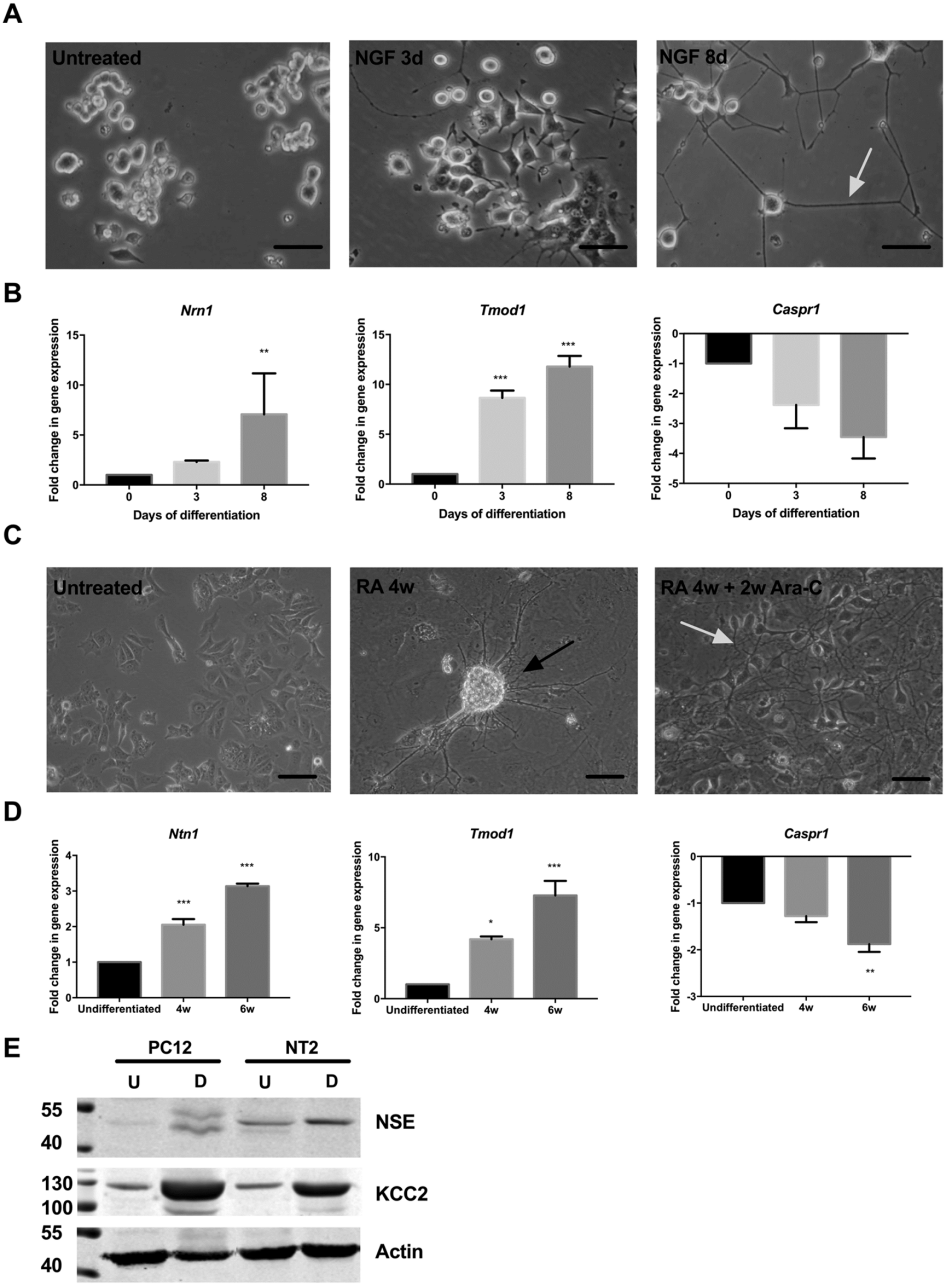
Characterising PC12 and NT2 morphology, gene and protein expression following differentiation into neuronal-like cells. **A:** Representative bright-field microscopy images of PC12 cell morphology after 3 and 8 days in the presence of NGF, (20x magnification). **B:** Relative *Nrn1*, *Tmod1* and *Caspr1* mRNA expression in differentiated PC12 cells was analysed by qPCR. **C:** Representative bright-field microscopy images of NT2 cell morphology were taken 4 weeks after treatment with all-trans-retinoic acid (ATRA 4w) and a further 2 weeks after treatment with cytosine arabinoside (Ara-C) (ATRA 4w + 2w Ara-C); (20x magnification). **D:** Relative *Nrn1*, *Tmod1* and *Caspr1* mRNA expression in differentiated NT2 cells was analysed by qPCR. **E:** Representative immunoblot analysis showing NSE and KCC2 protein expression in undifferentiated (U) and differentiated (D) PC12 and NT2 cells. Equal protein loading was assessed by immunoblotting for actin. **A** and **C:** Scale bar represents 50 μm; white arrows indicate interlaced axon-like structures and black arrows indicate ganglion-like clusters. **B** and **D**: Data is presented as the mean ± SEM; n = 3; **p≤0.01, ***p≤0.001.

PC12 cells were treated with 200 nM NGF and differentiation into neuronal-like cells was assessed morphologically. NGF treatment of PC12 cells rapidly initiated signs of differentiation. Untreated cells presented with round cell bodies, however after 3 days NGF treatment, PC12 cells developed neurite-like projections (Fig 1A). After 8 days, PC12 cells displayed a typical neuronal-like morphology with long projections and interlaced axon-like structures (Fig 1A, white arrow).

The relative expression of neuronal markers during PC12 cell differentiation was assessed by qPCR. *Neuritin 1 (Nrn1)* and *tropomodulin 1 (Tmod1)* expression was significantly increased (*p* = 0.004 and *p*<0.001 compared to untreated cultures) in PC12 cells after 8 days of NGF treatment (Fig 1B). By contrast, *contactin associated protein 1 (Caspr1)* expression was downregulated (*p* = 0.4331) by NGF treatment (Fig 1B). As *Nrn1*, and *Tmod1* are characteristically expressed in neurons[26,27], their upregulation supports the morphological neuronal-like changes in PC12 cells (Fig 1A).

Human NT2 cells differentiate into neuronal-like cells following all-trans-retinoic acid (ATRA) treatment[28]. NT2 cells started to produce axonal-like structures one week after ATRA treatment (data not shown), and these structures became thicker and longer after extended culture in ATRA (Fig 1C). NT2 cells also displayed longer projections and a typical neuronal morphology; ganglion-like clusters (Fig 1C, central panel, black arrow) and interlaced axon-like structures (Fig 1C, right panel, white arrow), as observed in primary neuronal cultures[29]. A homogenous population of neuronal-like cells was produced by sub-culturing NT2 in the presence of mitotic inhibitor, cytosine β-D-arabinofuranoside (C-Ara), to remove non-differentiated cells.

*Nrn1*, *Tmod1* and *Caspr1* expression was also assessed in NT2 cells (Fig 1D). Like PC12 cells, expression of *Nrn1* and *Tmod1* significantly increased (*p*<0.001) in NT2 cells during differentiation (Fig 1D). By contrast, *Caspr1* expression was significantly downregulated (*p* = 0.004) by ATRA treatment (Fig 1D). These results support the observed phenotypic changes in NT2 (Fig 1C) and are characteristic of neuronal differentiation [30,31].

Given the current interested in the chloride co-transporters (CCC) and their potential role in neuronal adaptation to ischaemic stroke [32], expression of neuron-specific enolase (NSE)[33] and the neuronal specific chloride co-transporter, KCC2 [34], were analyzed by immunoblotting (Fig 1E). Low basal KCC2 and NSE expression was detected in both PC12 and NT2 cells (Fig 1E) and a striking increase in expression of both markers was observed upon differentiation, particularly KCC2. Together, these data indicate differentiation of PC12 and NT2 cells induces changes in morphological, gene and protein expression, consistent with a neuronal-like phenotype.

### Determining PC12 and NT2’s response to hypoxia

**Fig 2:**
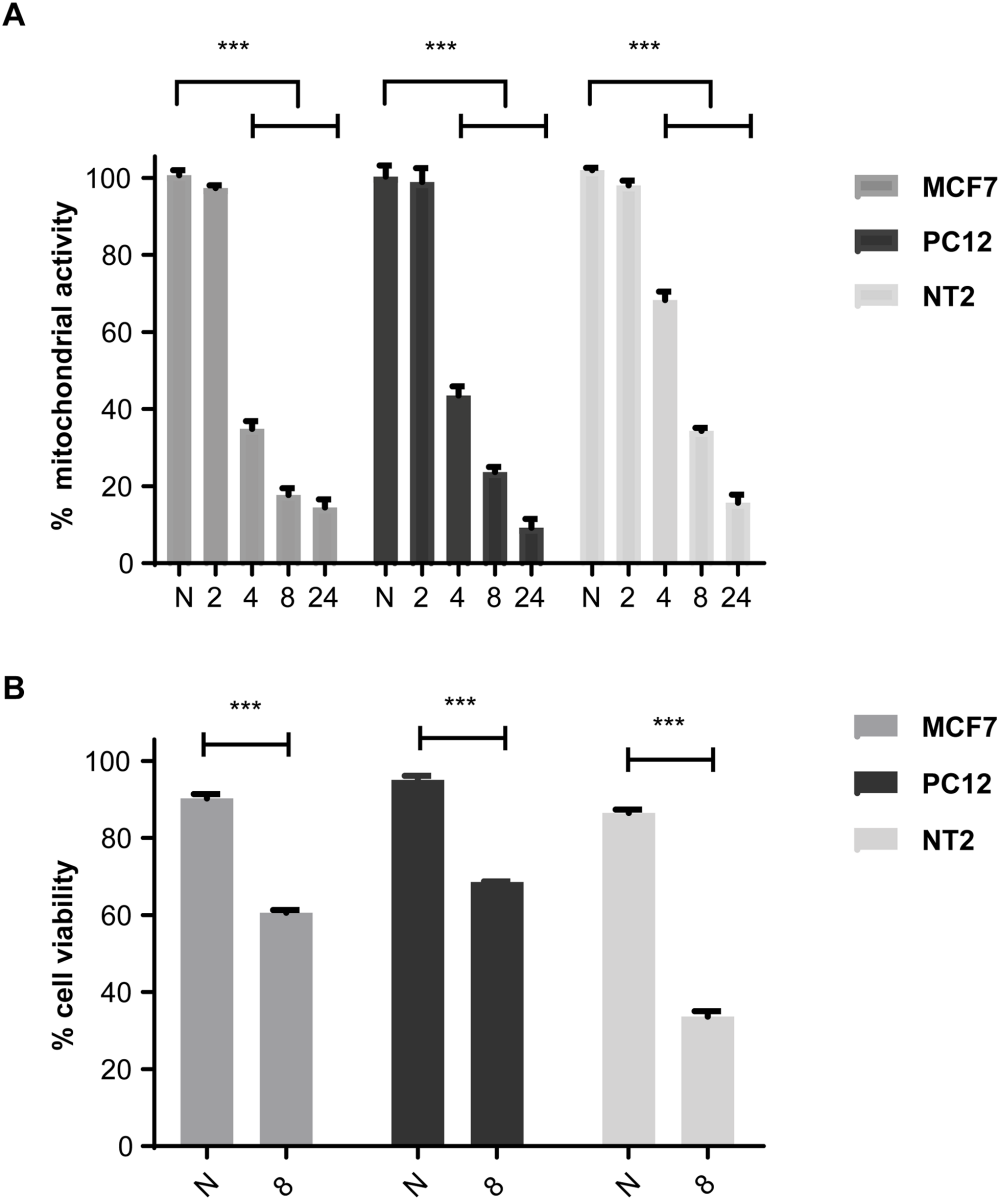
The effect of hypoxia on neuronal-like PC12 and NT2 cells. **A:** MCF7, PC12 and NT2 mitochondrial activity was analysed using alamarBlue 2, 4, 8 and 24 hours after exposure to hypoxia and expressed as a percentage of normoxic cell activity. **B:** MCF7, PC12 and NT2 cell viability was analysed via trypan blue staining 8 hours after exposure to hypoxia (8) and compared to staining of normoxic (N) cells. Data is expressed as mean ± SEM; n = 3; ***p≤0.001.

Mitochondrial activity was analysed in differentiated PC12 and NT2 cells, 2, 4, 8 and 24 hours after hypoxia via alamarBlue to determine a suitable exposure time for hypoxia (Fig 2A). Induction of hypoxia and the hypoxic response is well characterised in MCF7 cells [35,36], so these were included as a positive control throughout this study to verify acute hypoxic conditions were achieved. Little reduction in activity was observed 2 hours post-hypoxia, however a dramatic reduction in activity (*p*<0.001) was observed after 4 hr in all cell lines (Fig 2A). PC12 and MCF7 cells showed an intense decrease in activity at 4, 8 and 24 hr (Fig 2A), whilst NT2 cells were more resistant to hypoxic insult at these time points (Fig 2A).

The effect of acute hypoxia on viability of differentiated PC12 and NT2 cells was also assessed by their ability to exclude trypan blue dye. Eight hours of acute hypoxia significantly decreased PC12, NT2 and MCF7 cell viability (*p*<0.001); using this method, NT2 cells were more sensitive than PC12 cells to hypoxic insult (Fig 2B). Longer exposure to acute hypoxia (24 hours) resulted in a large number of detached cells (data not shown). Therefore, eight hours hypoxic insult was used in all subsequent experiments (unless otherwise stated) as it maintained sufficient viable cellular material for analysis. Detached cells were removed and only viable cells were analysed.

### Increased HIF-2α stability is observed in hypoxic neuronal-like PC12 and NT2 cells

**Fig 3:**
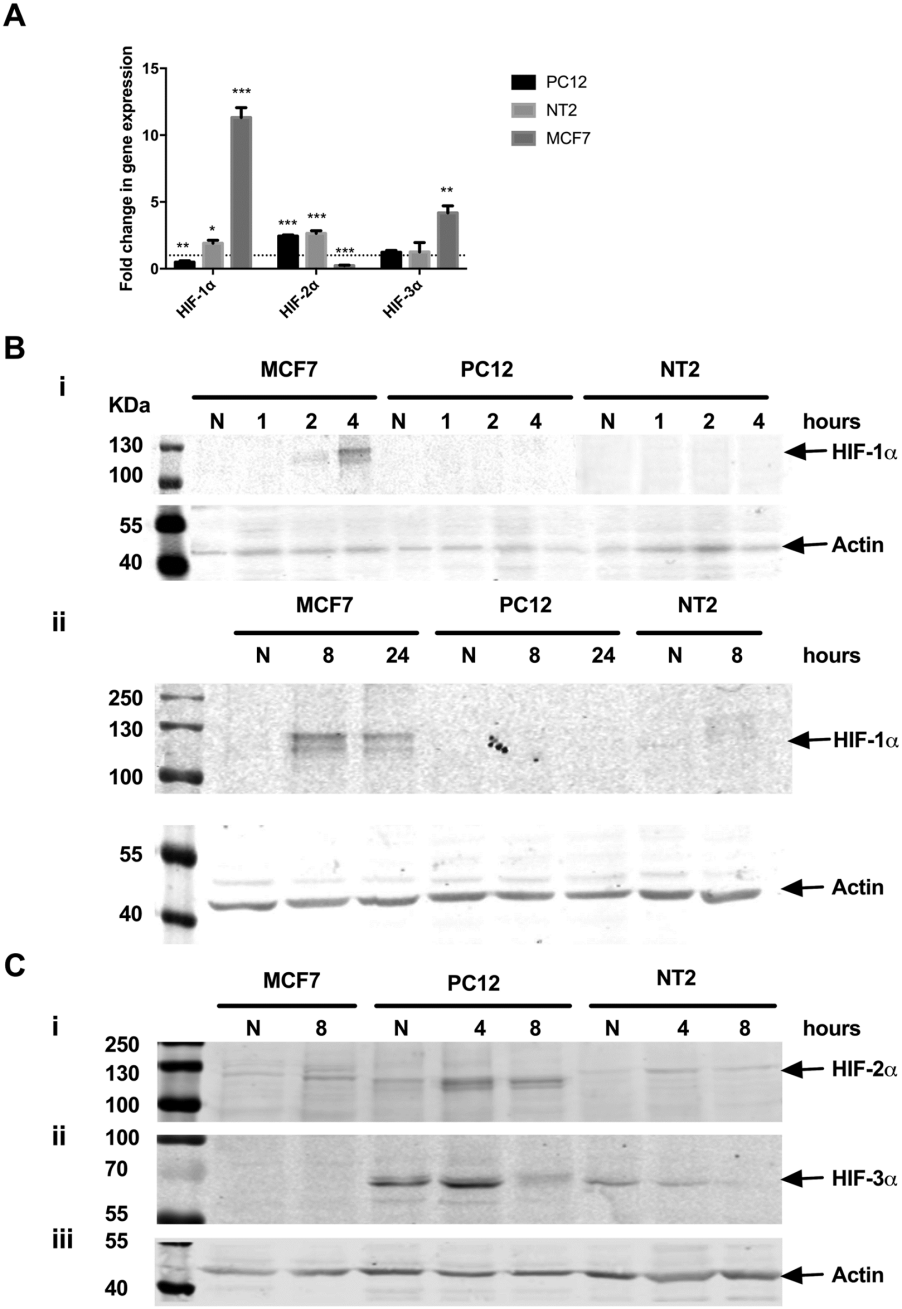
Increased HIF-2α stability is observed in neuronal-like PC12 and NT2 cells following hypoxia. **A:** Relative *HIF1-3α* mRNA expression was analysed in MCF7 and differentiated PC12 and NT2 cells exposed to 8 hours of hypoxia by qPCR. Data is expressed as mean ± SEM; n = 3; *p<0.05, **p≤0.01, ***p≤0.001. The dotted line represents basal gene expression. **B-C:** Representative immunoblots of HIF-1α (**B**), HIF-2α (**Ci**), HIF-3α (**Cii**) protein expression in MCF7 and differentiated PC12 and NT2 cells, 1, 2, 4, 8 or 24 hours after exposure to hypoxia. **B** and **C**: Equal protein loading was assessed by immunoblotting for actin.

Neuronal-like PC12 and NT2 cell adaptation to acute hypoxic stress was investigated by assessing *HIF-1α*, *HIF-2α* and *HIF-3α* gene expression. MCF7 cells were included as a positive control to verify acute hypoxia was successfully induced. *HIF-1α* mRNA expression was detected in all three cell lines, however a significant reduction in *HIF-1α* expression (*p* = 0.008) was observed in PC12 cells following hypoxia, and only a small increase in expression (*p* = 0.02) was observed in NT2 cells (Fig 3A). By contrast, and in keeping with work by others [35], MCF7 cell displayed a dramatic 11.3-fold up-regulation of *HIF-1α* mRNA (*p*<0.001), 8 hr after hypoxia (Fig 3A). Hypoxia triggered a significant increase (*p*<0.001) in *HIF-2α* mRNA expression in differentiated PC12 and NT2 cells (Fig 3A, 2.4- and 2.6- fold respectively) whilst *HIF-2α* expression in hypoxic MCF7 cells was significantly decreased (*p*<0.001). *HIF-3α* mRNA showed relatively stable levels of expression in PC12 and NT2 cells and was relatively unchanged in response to hypoxia (Fig 3A). By contrast, *HIF-3α* mRNA was upregulated (*p* = 0.0037) in MCF7 cells by hypoxic insult (Fig 3A).

**Fig 4:**
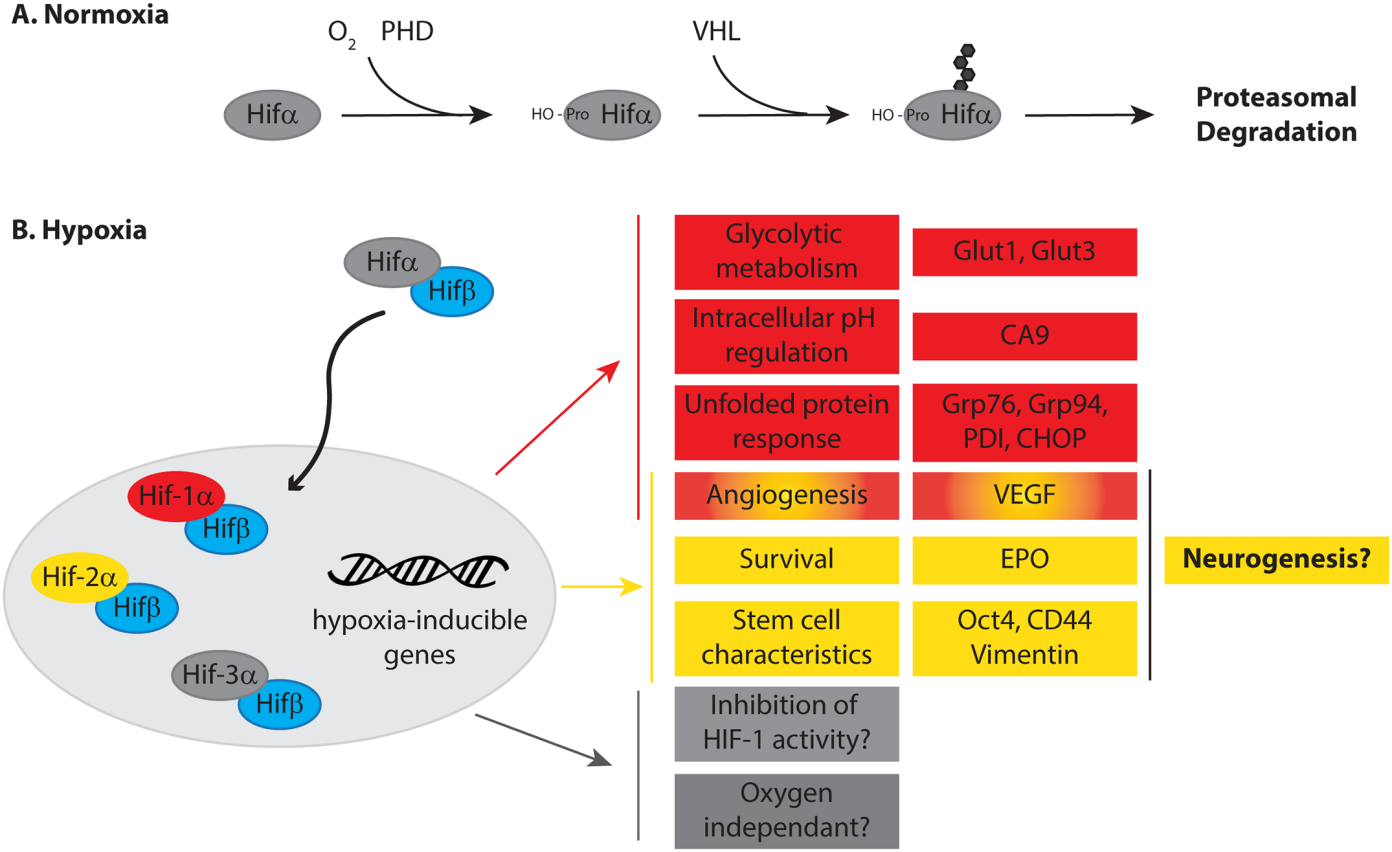
HIF mediated adaptation to hypoxic stress. **A:** Under physiological oxygen concentrations, HIF-1α/2α are hydroxylated by prolyl hydroxylases (PHD), promoting HIF-1α/2α binding to the E3 ligase, von Hippel-Lindau protein (pVHL), and their ubiquitination [3]. This maintains very low basal expression of HIF-1α/2α due to rapid proteasomal degradation. **B:** Under hypoxic conditions, PHD activity is inhibited [38]. This stabilises HIF-1α/2α expression, enhancing binding to HIFβ and translocation to the nucleus and transcription of various hypoxia-responsive genes[3]. HIF-1α and -2α share regulation of several genes, yet also regulate distinct subsets [4,6]. HIF-3α function is not yet fully understood. Abbreviations: CHOP, CCAAT-enhancer-binding protein homologous protein; EPO, Erythropoietin; Grp, Glucose regulated protein; HIF: Hypoxia Inducible Factor; PDI, protein disulphide isomerase; PHD, Prolyl hydroxylase; VEGF, Vascular epithelial growth factor; VHL, Von Hippel Lindau.

HIF-1α protein undergoes post-translational stabilisation in response to hypoxic stress [37] (summarised in Fig 4). HIF-1α expression was analysed by immunoblotting using commercially available antibodies recognising distinct HIF-1α epitopes (400–550 aa and 610–727 aa). A variety of conditions and antibody dilutions were tested however, HIF-1α induction in response to hypoxia was not detected in differentiated PC12 at any of the time points analysed (Fig 3B) and only a very slight increase in HIF-1α expression was detected in differentiated NT2 cells. By contrast, HIF-1α induction was clearly detected in MCF7 cells (Fig 3B, panel i and ii) after 2 hours of hypoxia and reached a maximum induction after 4–8 hours (Fig 3B, panel ii). HIF-1α induction in MCF7 verifies the validity of the experimental conditions and antibodies used here.

Bands corresponding to HIF-2α were readily detected in PC12, NT2 and MCF7 cells and their intensity increased after 4 hours of hypoxia (Fig 3C, panel i). HIF-3α expression was evident in both neuronal-like cell lines under normoxic conditions (Fig 3C, panel ii) however, a differential response to hypoxia was observed between PC12 and NT2 cells. HIF-3α expression was initially induced by hypoxia in PC12 cells yet reduced below basal expression at 8 hours (Fig 3D). By contrast, the intensity of HIF-3α expression progressively decreased in NT2 cells after 4 hours of hypoxia (Fig 3D). HIF-3α expression was not detected in MCF7 cells under either normoxic or hypoxic conditions.

Together, these findings suggest that under the experimental conditions tested here, HIF-1α is not stabilised in response to acute hypoxia in the neuronal-like cell models examined. The observed induction of HIF-2α and HIF-3α mRNA and protein expression suggests neuronal-like cells may activate different arms of the hypoxic adaptive machinery and HIF-2α mediated signalling may predominate over HIF-1α signalling in hypoxic neuronal-like cells.

### Hypoxic adaption via HIF-2α predominates in neuronal-like cells

To further analyse potential differences in hypoxic adaptation in neuronal-like cells, regulators of *HIF-1α* and *HIF-1α* signalling were investigated. *Ptbp2* (poly pyrimidine track binding protein 2), binds the internal ribosome entry site in *HIF-1α*’s 5′-UTR, promoting efficient HIF-1α translation and up-regulation during hypoxia [39]. Carbonic anhydrase, isoform 9 (*Ca9*) contains a hypoxia-responsive element in its promoter [40] and is dramatically up-regulated following hypoxia via HIF-1α transcriptional regulation [41].

**Fig 5:**
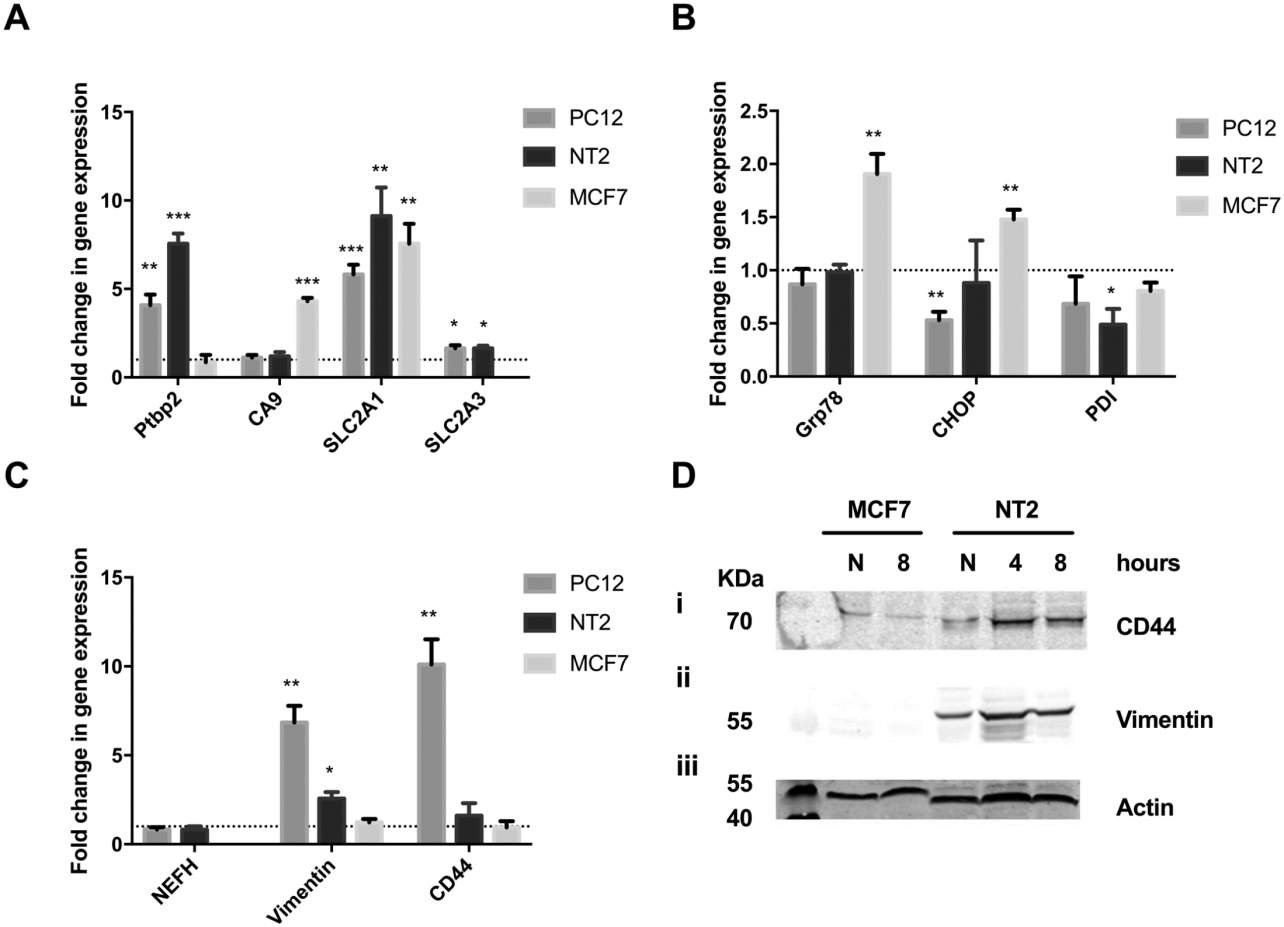
HIF-2α dependant pathways are preferentially activated in differentiated PC12 and NT2 cells following hypoxia. Relative expression of HIF-1α related target genes (*Ptbp2*, *CA9*, *SLC2A1* and *SLC2A3*) (**A**); UPR related genes (*Grp78*, *CHOP* and *PDI*) (**B**); and HIF-2α related target genes (*NEFH*, *Vimentin* and *CD44*) (**C**), were analysed in MCF7 and differentiated PC12 and NT2 cells, 8 hours after hypoxia, using qPCR. Data is expressed as mean ± SEM; n = 3; *p<0.05, **p≤0.01, ***p≤0.001. The dotted line represents basal gene expression. **D:** Representative immunoblots showing induction of CD44 (**i**) and vimentin (**ii**) protein expression in normoxic (N) or hypoxic (4 or 8 hours hypoxia) MCF7 and differentiated NT2 cells. Equal protein loading was assessed by immunoblotting for actin (**iii**).

*Ptbp2* and *Ca9* gene expression was analysed by qPCR (Fig 5A). Increased *Ptbp2* expression was observed in hypoxic neuronal-like PC12 (*p* = 0.0069) and NT2 cells (*p*<0.001), while little change in *Ptbp2* expression was observed in hypoxic MCF7 cells (Fig 5A). By contrast, *Ca9* expression remained relatively unchanged in hypoxic PC12 and NT2 cells (Fig 5A) yet was significantly upregulated in MCF7 cells (*p*<0.001). This is in keeping with HIF-1α dependant regulation of *Ca9* expression [41] and the observed lack of HIF-1α induction in neuronal-like cells (Fig 3).

*Slc2A1 and Slc2A3* encode the glucose transporters, GLUT1 and 3, and their expression is regulated by HIF-1α and HIF-2α [42]. *Slc2A1* expression was significantly increased in neuronal-like PC12 (*p* = 0.007) and NT2 cells (*p* = 0.004), and MCF7 cells in response to hypoxia (Fig 5A). A small but significant increase in *Slc2A3* expression was also detected in neuronal-like PC12 (*p* = 0.02) and NT2 cells (*p* = 0.01) following hypoxia (Fig 5A) however *Slc2A3* expression was not detected in MCF7 cells or induced by hypoxic insult (Fig 5A), in keeping with GLUT3’s status as a neuronal specific glucose transporter [43].

### Hypoxic neuronal-like cells are more resistant to ER stress

Hypoxic stress triggers accumulation of misfolded proteins in the endoplasmic reticulum (ER)[44]. The unfolded protein response (UPR) serves to redress ER homeostasis by fine-tuning protein translation and enhancing folding capacity [45]. Growing evidence suggests HIF and UPR dependent pathways interact to coordinate gene expression, metabolism and cell survival [46].

Expression of UPR markers, *Grp78*, *PDI* and *CHOP*, was measured via qPCR in neuronal-like PC12 and NT2 cells, 8 hours after hypoxic insult (Fig 5B). *Grp78* expression was unchanged by hypoxia in PC12 and NT2 cells yet was significantly increased (*p* = 0.0086) in MCF7 cells (Fig 5B). *CHOP* expression was also unchanged by hypoxia in NT2 cells yet was significantly reduced in hypoxic PC12 cells (*p* = 0.0039) and increased in hypoxic MCF7 cells (*p* = 0.0064) (Fig 5B). *PDI* expression was relatively unchanged in hypoxic PC12 cells but showed a small but significant reduction (*p* = 0.0260) in hypoxic NT2 cells (Fig 5B). Collectively, this suggests neuronal-like cells present a higher resistance to ER stress following acute hypoxia under our experimental conditions.

### Hypoxic adaption in neuronal-like cells drives expression of genes associated with a neuronal progenitor phenotype

Whilst HIF-1α and -2α share target genes, HIF-2α regulates gene expression independently of HIF-1α and promotes different aspects of hypoxic adaptation [6] (Fig 4). HIF-2α regulates expression of stem cell markers [47] and maintains the undifferentiated state, via expression of neural crest and stem cell-associated genes [48]. Vimentin and CD44 are highly expressed in neuronal precursor cells [49,50], however expression declines during development as neurons become post-mitotic. In post-mitotic neurons, vimentin is replaced by neurofilaments [51].

Expression of the neuronal marker, neurofilament heavy chain (*NEFH*) [52] was evident in differentiated PC12 and NT2 cells yet undetected in MCF7 cells (Fig 5C); basal expression was also unaffected by hypoxic insult in PC12 and NT2 cells. *Vimentin* expression was significantly upregulated in PC12 (*p* = 0.0033) and NT2 cells (*p* = 0.0119) following hypoxia (Fig 5C). *CD44* expression was also dramatically increased in hypoxic PC12 cells (*p* = 0.003), whilst expression was only modestly effected in hypoxic NT2 cells (1.6-fold increase; *p* = 0.43; Fig 5C). *Vimentin* and *CD44* expression were not significantly altered in hypoxic MCF7 cells (Fig 5C).

CD44 and vimentin expression was also analysed by immunoblotting. CD44 and vimentin expression were readily detected in normoxic NT2 cells and markedly induced by hypoxia (Fig 5D). CD44 expression was unchanged in hypoxic MCF7 cells (Fig 5D), however vimentin was not detected in MCF7 cells in either normoxic or hypoxic conditions (Fig 5D). Together, these findings show strong induction of HIF-2α regulated genes in hypoxic neuronal-like cells and suggest the HIF-2α dependant cellular regeneration arm of the adaptive hypoxic response predominates over the HIF-1α arm in neuronal-like cells following acute hypoxia.

### Hypoxia promotes a regression to undifferentiated states in neuronal-like cells

**Fig 6:**
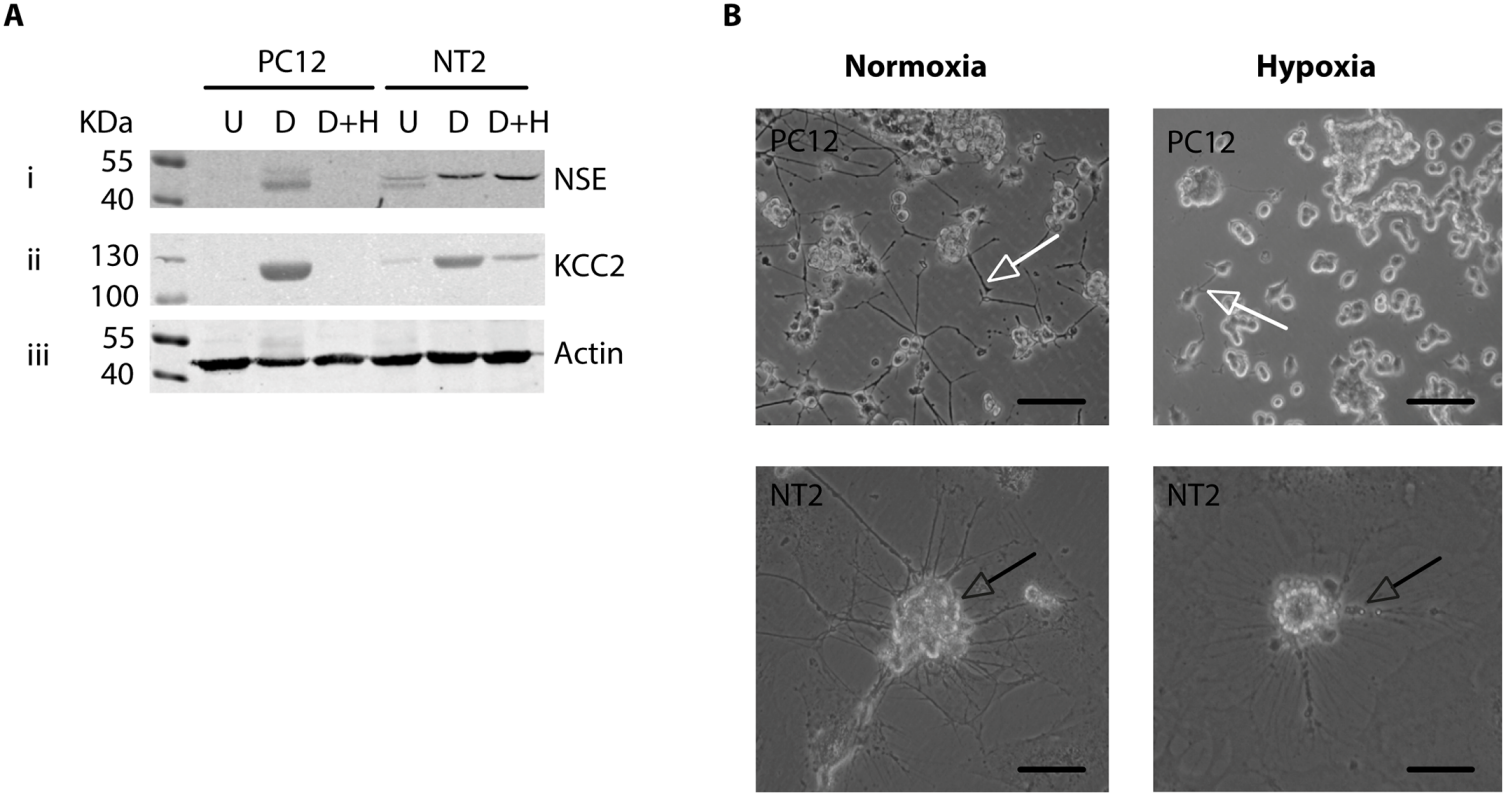
Hypoxia promotes a regression of neuronal-like cells to undifferentiated states. **A:** Representative immunoblots showing the change in NSE (**i**) and KCC2 (**ii**) protein expression in normoxic (N), differentiated (D) or differentiated and hypoxic (D+H, 8 hours) PC12 and NT2 cells. Equal protein loading was assessed by immunoblotting for actin (**iii**). **B:** Representative bright-field microscopy images of differentiated PC12 and NT2 morphology after exposure to 8 hours of normoxia or hypoxia (20x magnification). Scale bar represents 50 μm. White arrows indicate interlaced axon-like structures and black arrows indicate ganglion-like clusters.

Neuron specific markers, NSE and KCC2 were dramatically induced following neuronal differentiation of PC12 and NT2 cells (Fig 1E). NSE and KCC2 expression was assessed by immunoblotting to determine whether hypoxia could reverse the expression of these neuronal markers. The dramatic increase in NSE expression was attenuated following hypoxia in differentiated PC12 cells, whereas NSE expression remained unchanged by hypoxia in differentiated NT2 cells (Fig 6A, panel i). The striking induction of KCC2 expression observed following differentiation was also dramatically reversed in hypoxic PC12 cells after as little as 8 hours of hypoxic insult and severely reduced in hypoxic NT2 cells (Fig 6A, panel ii).

Differentiated PC12 and NT2 display neurite-like processes and gangliar-like structures (Figs 1F and 6B). The neuronal-like morphology was dramatically modified in as little as 8 hours of hypoxic insult (Fig 6B); the number of differentiated PC12 and NT2 cells presenting with neurites was dramatically reduced and the length of neurites was evidently shortened. These results combined with the observed HIF-2α dependant induction of neural progenitor cell markers (Fig 5B and 5D) suggest adaptations promoted by HIF-2α following acute hypoxic insult could drive neuronal-like cells into a more immature phenotype.

## Discussion

Stroke is the second leading cause of death and disability worldwide [53]. PC12 and NT2 cells have been extensively used as models to study neuronal differentiation and neurological pathologies, including stroke [54–56]. Furthermore, NT2 cells are currently undergoing phase 1 and 2 clinical trials as neuronal grafts for stroke patients [57,58]. Whilst these cells are not primary neurons, differentiated PC12 and NT2 cells are a valid and commonly used source of neuronal-like cells, and an excellent model to study the hypoxic molecular adaptations that occur *in vivo*, following stroke.

Numerous studies have investigated the role of HIF-1α in neuronal adaptation to hypoxia [59,60] (Fig 4). HIF-2α and HIF-3α signalling have received less attention, however, recent work demonstrates HIF-2α is an essential component of hypoxic adaptation [6]. Likely, these pathways are complimentary and co-ordinated signalling of both HIF-1α and HIF-2α is required to promote survival and adaption following hypoxic insult (Fig 4). To our knowledge, this is the first study to dissect the importance of HIF dependant adaptation and downstream signalling in differentiated neuronal-like cells following hypoxia.

HIF-1α expression and induction following hypoxia has been reported in PC12 and NT2 cells [61,62] and neuroblastoma cells [59], therefore our observed lack of HIF-1α induction and signalling following acute hypoxia, as well as through chemical induction of hypoxia, using colbalt chloride (data not shown), was surprising. However, closer scrutiny reveals fundamental methodological differences with these studies which may account for the absence of HIF-1α induction, such as employing undifferentiated cells [62,63], culturing cells on uncoated plates [64], using different exposure times [65] or oxygen concentrations that confer mild hypoxia [62]. Interestingly, we observed consistent, preferential induction of HIF-2α over HIF-1α expression in differentiated PC12 and NT2 cells when acute hypoxia was induced, for 8 hours, with slightly different gas mixtures (1% O_2_/99% N_2_ and 1% O_2_/5% CO_2_/99% N_2_, data not shown). Our model utilises fully differentiated post-mitotic neuronal-like cells, and is therefore potentially more representative of neuronal cell function than undifferentiated cells.

Despite the lack of HIF-1α induction in PC12 and NT2 cells, acute hypoxic conditions were achieved. MCF7 cells were used as a positive control as they show intense HIF-1α expression following acute hypoxia [35,36]. Hypoxia was induced simultaneously with NT2 and PC12 cells and rapid HIF-1α stabilisation and intense activation of *HIF-1α* signalling was observed in control MCF7 cells, confirming acute hypoxia was established. Marked induction of *Ptbp2 and Slc2A1* was observed in hypoxic PC12 and NT2 cells; genes thought to be specifically regulated by HIF-1α [66]. However, many HIF-1α specific genes have also been shown to be regulated by HIF-2α, including *Slc2A1* (GLUT-1)[67], underscoring the potential importance of HIF-2α signalling in hypoxic adaptation of neuronal-like cells.

Surprisingly, neuronal-like PC12 and NT2 cells are refractory to HIF-1α stabilisation in response to acute hypoxia and activation of HIF-1α signalling was absent. Instead, significant induction of *HIF-2α* mRNA and stabilisation of HIF-2α and HIF-3α protein was evident in hypoxic PC12 and NT2 cells. HIF-1α, HIF-2α expression is detected in undifferentiated neuroblastoma cells [59] however HIF-1α expression appears to be dominant and HIF-2α induction was only observed in certain cells under complete anoxia (0% O_2_). These findings suggest the HIF-2α arm of the hypoxic adaptive response may predominate over HIF-1α dependant mechanisms in differentiated neuronal-like cells and underscore the need to use differentiated neuronal *in vitro* models as a platform to investigate the molecular changes occurring following ischaemic stroke.

HIF-2α regulates several genes involved in proliferation and regeneration, including the *OCT4* transcription factor which maintains stem-like characteristics [68] and neural stem cell pluripotency [69,70]. HIF-2α signalling also maintains cells in an undifferentiated state. Expression of neural crest genes is increased by hypoxia, and may drive a more immature phenotype in neuroblastoma cells [59]. Increased expression of the stem cell marker, CD44 and early neuronal progenitor marker, vimentin, was readily observed in hypoxic neuronal-like cells and were accompanied by loss of neuronal markers, NSE and KCC2, and neuronal morphology. HIF-2α dependant hypoxic adaptation therefore may promote regression of neuronal-like cells to a more undifferentiated, and thus potentially transient, proliferative, state prior to repair. In doing so, HIF-2α signalling may have some utility in triggering neuronal repair, recovery and regeneration following acute hypoxic insult (Fig 4). Indeed exogenous administration of the HIF-2α target, EPO, can promote angiogenesis and neurogenesis following neonatal ischaemia in rats [71]. Preferentially activating the HIF-2α dependant arm of the hypoxic adaptive response could therefore represent a novel strategy to minimise damage associated with ischaemic stroke and promote neural repair and regeneration.

In the adult brain, neurogenesis occurs in the subventricular and hippocampal subgranular zones and to a lesser extent, the striatum and cerebral cortex [72]. Neural stem cell proliferation is dramatically increased following stroke [51,73] and progenitor cells can migrate to sites of infarct [74], however the vast majority of these immature neurons fail to survive, likely due to unfavourable environmental conditions or lack of functional connections [51,75]. Transient hypoxia can increase the number of mitotic neurons and expression of proliferative markers in cultured embryonic rat cortical neurons [76] whilst mild hypoxia favours proliferation and differentiation of neural spheres [77]. Together, these studies suggest sub-lethal hypoxic insult could potentially promote self-renewal *in vivo*. In the heart, chronic stabilisation of HIF-1α and HIF-2α protects against ischaemia reperfusion injury in adult mice [78] and systemic hypoxia promotes cardiomyocyte proliferation [79]. This suggests hypoxic stress may also act as a driving force for proliferation and a potential therapeutic tool in regenerative medicine.

The role of HIF in the pathophysiology of ischaemic stroke and its impact on neuronal survival remains controversial. *In vivo* global and focal ischaemia models provide conflicting results as genetic manipulation of neuronal *HIF-1α* has been reported to be both neuroprotective and detrimental [10,80]. HIF-1α overexpression has also been associated with dendritic overgrowth and abnormal patterning of cortical neurons [81]. Activation of HIF-1α dependant signalling may therefore be potentially deleterious to neurons in ischaemic stroke. Combined loss of HIF-1α and HIF-2α has been found to be detrimental for functional recovery after ischaemic stroke but surprisingly beneficial in the early stroke phase [12]. This suggests partial compensatory mechanism exist between the two transcription factors.

Ischaemic neuronal injury is a multifunctional process [1,2]. Spatial and temporal factors combined with the intensity of ischaemic challenge will all impact upon HIF dependant adaptation and whether downstream signalling promotes neuroprotection, ischaemia induced cell death or neuroproliferation. These areas require significantly greater research and specific therapeutic agents targeting HIF-1α and HIF-2α are needed to delineate the intricacies of HIF signalling and understand their therapeutic potential.

Due to the potential of small populations of neural stem cells to regenerate neuronal tissue and drive repair and recovery of damaged regions of brain tissue, understanding the mechanisms involved in promoting self-repair and how non-neurogenic areas in the brain can revert to neural stem/progenitor cells is crucial. Our results show the HIF-2α arm of the hypoxic adaptive response may predominate over the HIF-1α arm in neuronal-like cells and suggest this could promote regression to a more dedifferentiated state. Whilst caution must be taken in extrapolating *in vitro* findings to an *in vivo* setting, it is possible that preferential activation of HIF-2α dependant adaptations in the small population of neuronal cells surviving the transient hypoxia of a stroke could drive expression of stem-like genes and promote the dedifferentiation of these cells. These dedifferentiated cells could then promote the self-renewal and growth of neuronal cells in the hours to days following a stroke. Ultimately, this could promote the repair of damaged neuronal tissue and reduce the long-term damage associated with ischaemic insult. Therapeutic manipulation of endogenous pathways driving neuronal adaptation to hypoxic stress could represent exciting possibilities to enhance repair and recovery of the stroke damaged brain and promote neurogenesis, and ultimately reduce stroke-associated disability.
